# Understanding the haplotypic diversity and distribution of malaria-protective β-globin mutations

**DOI:** 10.1186/1475-2875-13-S1-P42

**Published:** 2014-09-22

**Authors:** Carinna Hockham, Bridget Penman, Frédéric Piel, Sunetra Gupta

**Affiliations:** 1 Department of Zoology, University of Oxford, Oxford, UK

## Background

Sickle cell (HbS) and β^0^-thalassaemia, two common malaria-protective β-globin mutations, represent impressive examples of human adaptation to infectious disease. Highly prevalent in Sub-Saharan Africa and the Mediterranean, respectively, these mutations display contrasting patterns in their haplotype structure and distribution. The majority of HbS is associated with five major restriction fragment length polymorphism patterns that exhibit remarkable geographical specificity. By contrast, β^0^-thalassaemia, which can result from several different mutations, is found on many more haplotypes whose distributions considerably overlap. Here, we shed light on the evolutionary and demographic factors that likely gave rise to such differences, despite malaria being a common selective force.

## Methods

A stochastic population genetic model, which also incorporated individual-based processes, was developed to simulate and track the evolution of HbS and β^0^-thalassaemia haplotypes, separately, in a meta-population. Under no a priori assumptions, the influence of mutation, gene conversion, fitness variation and population structure on the spread of individual mutant β-globin haplotypes through a network was investigated.

## Results

The outcomes resembling the present-day patterns of HbS and β^0^-thalassaemia haplotypes were obtained under somewhat restrictive, and indeed distinct, parameter combinations. For both mutations, we consistently observed allelic exclusion, whereby subpopulations already containing one haplotype at a high frequency were rarely taken over by another. This has several implications. First, the formation of the present-day HbS haplotype pattern requires minimal gene flow and strong population structure such that multiple alleles can arise and spread in distinct regions of the meta-population without interference from each other. By contrast, the overlapping of haplotypes, as for β^0^-thalassaemia, was more common when movement through the network was less restricted to allow separately dispersing haplotypes to meet when they are still at low frequency. Finally, as gene conversion can occur only in subpopulations already polymorphic for a mutation, haplotypes arising through gene conversion are frequently excluded unless fitness is sufficiently varied.

## Conclusion

We have developed a generic framework to explore the processes affecting the evolution and spread of genetic diseases, and applied it to the study of the haplotype evolution of two malaria-protective disorders. In doing so, we have been able to delineate the conditions under which the observed present-day patterns of HbS and β^0^-thalassaemia haplotypes are most probable, and identified the major processes contributing, under malaria selection, to their haplotypic evolution. The model is highly versatile and can be used to explore both basic evolutionary biology questions and applied public health enquiries.

